# Insoluble Fiber in Barley Leaf Attenuates Hyperuricemic Nephropathy by Modulating Gut Microbiota and Short-Chain Fatty Acids

**DOI:** 10.3390/foods11213482

**Published:** 2022-11-02

**Authors:** Yongmei Li, Lu Li, Jinhong Tian, Fengxin Zheng, Hui Liao, Zean Zhao, Yanyu Chen, Jianxin Pang, Ting Wu

**Affiliations:** Guangdong Provincial Key Laboratory of Drug Screening, School of Pharmaceutical Sciences, Southern Medical University, Guangzhou 510515, China

**Keywords:** barley leaf, hyperuricemic nephropathy, gut microbiota, SCFAs, URAT1, GLUT9

## Abstract

Hyperuricemia (HUA), characterized by abnormal serum uric acid (UA) levels, is recognized as an important risk factor for hyperuricemic nephropathy (HN), which is strongly linked to gut microbiota. This study investigated the protective effects and regulatory mechanisms of insoluble fiber from barley leaves (BL) against HN, induced by adenine (Ad) and potassium oxonate (PO). The results showed that BL dramatically reduced the levels of serum UA and creatinine (CR) and alleviated renal injury and fibrosis. Moreover, BL modulated oxidative stress and downregulated the expression of urate transporter 1 (URAT1) and glucose transporter 9 (GLUT9) in the kidneys of mice with HN. In addition, the 16S rRNA sequence data showed that BL also increased the relative abundance of short-chain fatty acids (SCFAs)-producing bacteria, including *Bacteroides*, *Alloprevotella*, and *Eisenbergiella*. Besides, BL treatment also increased SCFAs levels. Of interest, the application of SCFAs in hyperuricemic mice effectively reduced their serum UA. Furthermore, SCFAs dose-dependently inhibited URAT1 and GLUT9 in vitro and potently interacted with URAT1 and GLUT9 in the docking analysis. When taken together, our results indicate that BL and its metabolite SCFAs may be potential candidates for relieving HUA or HN.

## 1. Introduction

Hyperuricemia (HUA) is recognized as a metabolic disorder with abnormally increased serum uric acid (UA). Elevated UA levels may form into urate crystals and deposit in the joints, thus causing gout or hyperuricemic nephrology (HN) [1,2]. HN is characterized by renal tubular injury, tubulointerstitial fibrosis, and glomerulosclerosis. Many studies have addressed that the renal damage caused by UA is mainly mediated through oxidative stress, renal inflammation, endothelial-to-mesenchymal transition, and so on [3,4]. Therefore, lowering serum UA levels and protecting the kidneys from damage are two important ways to treat HN.

UA, the final product of purine metabolism in the body, is mainly produced in the liver by the catalysis of xanthine oxidase. UA homeostasis involves complex physiological processes involving several organs, including the livers, kidneys, and intestines [5]. The kidney and intestine both take charge of the excretion of UA from the body. The kidneys account for approximately 75%, while the intestines account for the remaining 25% [6,7]. Clinically, 90% of patients with HUA are under-excreting [8,9]. UA excretion depends on various UA transporters. The critical transporters, like urate transporter 1 (URAT1) and glucose transporter 9 (GLUT9), are in charge of UA reabsorption, while ATP-binding cassette superfamily G member 2 (ABCG2) and organic anion transporter 1/3 (OAT1/3) are responsible for UA secretion [10,11]. Notably, URAT1 and GLUT9 are currently popular targets for developing UA-lowering drugs [12]. Clinically, the uricosuric drugs probenecid and benzbromarone promote UA excretion by reducing UA reabsorption via URAT1 [13]. However, due to the low selectivity of probenecid and the susceptibility of benzbromarone to fulminant hepatitis, the clinical applications of probenecid and benzbromarone are limited [14].

It is well known that dietary factors are closely associated with HUA and gout. Thus, dietary intervention is an important way to prevent HUA [15]. Recently, nutrition surveillance research [16] among Chinese adults and a US survey [17] suggested that the intake of food that is rich in grain fiber and total dietary fiber was significantly negatively correlated with the increasing occurrence risk of HUA. Dietary fiber is a type of polysaccharide widely found in grains, vegetables, and soybeans and is an integral part of a healthy diet. Dietary fiber can be fermented by the microorganisms in the colon and can alter gut microbiota composition, accompanied by the production of some active metabolites like short-chain fatty acids (SCFAs), such as acetate, propionate, and butyrate [18]. Differences in the composition of gut microbiota were detected between healthy individuals and patients with HUA [19,20,21]. A recent study revealed that the abundance of SCFAs-producing bacteria decreased significantly in *Uox*-knockout mice with HUA, leading to reduced SCFAs levels [22]. Interestingly, dietary interventions such as probiotics or prebiotics were demonstrated to significantly improve HUA by affecting intestinal flora and enhancing SCFAs levels [23,24,25]. However, the direct effects of SCFAs on UA metabolism have been under-investigated.

Barley leaf (BL), the young grass of barley (*Hordeum vulgare* L.), is consumed as a kind of popular healthy beverage in Japan and China. BL is found to be rich in insoluble fibers [26], which have been proven to enhance immunity, regulate blood pressure, control blood glucose levels, and so on [27]. Li [28] found that supplementation with BL in mice resulted in alterant gut microbiota composition and the production of microbial-associated metabolites. They also reported that BL exerted antioxidative and anti-inflammatory effects on colitis, which might be associated with its regulation of gut microbiota and SCFAs levels [29,30,31]. In addition, BL was also reported [32] to reduce serum UA in humans with HUA. However, the potential mechanisms have not been thoroughly investigated. In the present study, we explored the protective properties of BL on HN and also explored the pivotal roles of its metabolites SCFAs in UA metabolism.

## 2. Materials and Methods

### 2.1. Drugs

Potassium oxonate (PO), adenine (Ad), hypoxanthine (Hx), acetic acid, propionic acid, butyric acid, sodium acetate, sodium propionate, and sodium butyrate (all of the purity ≥ 98%) were brought from Aladdin (Shanghai, China). ^14^C-urate (55 mCi/mmol) was provided by American Radiolabeled Chemicals, Inc. (St. Louis, MO, USA). Uric acid (UA) was acquired from Sigma–Aldrich (St. Louis, MO, USA).

### 2.2. Preparation of Barley Leaf Powder

Barley leaf (BL) powder was provided by Hebei Biotechnology Co., Ltd. (Jiaxing, China). The preparation process was as previously described [28]. In brief, the fresh leaves of *Hordeum vulgare* L. were harvested when the barley grew to 20–30 cm and then was washed and crushed into a 1000 mesh powder by wall-broken technology. The powder was dried in a freeze dryer, pulverized with a blender, and sieved. The proximate analyses of the powder used in this study are shown in Table S1.

### 2.3. In Vivo Experimental Design

Male Kunming mice (18–22 g) were originally purchased from the Experimental Animal Center of Southern Medical University (Guangzhou, China). They were adapted under controlled conditions in a constant 12 h light/dark cycle SPF animal facility for one week and were free to access feed and water during the study. The whole study was approved by the Ethics Committee of Southern Medical University (approval date: 15 January 2020).

#### 2.3.1. Experiment 1: Treatment of BL in Mice with HN

A total of 24 mice were randomly assigned to four groups, including the control group (Con), BL-treated control group (BL), hyperuricemic nephropathy model group (HN), and BL-treated hyperuricemic nephropathy group (HN + BL), with six mice in each group. During the experiment, the Con group and HN group were invariably fed a standard chow diet. From the 1st day to the 14th day of the experiment, the two BL treatment groups were changed to an isocaloric diet containing 2.5% BL, which contained approximately 1.34% insoluble fiber (shown in Table S2), as the previous study described [29]; a dosage was calculated according to a previous human intervention study [33]. Meanwhile, the food intake was recorded every three days. During the BL intervention, the mice in the HN group and HN + BL group were induced to HN at the same time by receiving PO (350 mg/kg/d BW) intraperitoneally and Ad (70 mg/kg/d BW) by gastric gavage at 9:00 am, per day, for 14 days, as the previous study described [34]. The Con group and the BL group received the vehicle (0.5% CMC-Na). On the 13th day, all were placed in metabolic cages separately to obtain their urinary and fecal samples. On the 14th day, the animals were anesthetized with 5% chloral hydrate by intraperitoneal injection 90 min on the last day. Then, blood samples and kidneys were collected for further analysis.

#### 2.3.2. Experiment 2: Treatment of SCFAs in Mice with HUA

In the study of the SCFAs treatment, 36 mice were randomly allocated into six groups, including the control group, HUA model group, sodium acetate (75 mM, 0.2 mL) + HUA group, sodium propionate (75 mM, 0.2 mL) + HUA group, sodium butyrate (75 mM, 0.2 mL) + HUA group, and the RDEA3170 (5 mg/kg) + HUA group. RDEA3170 was used as the positive control. PO and Hx were applied to establish the HUA model in the mice, as previously described by us [35]. SCFAs were given orally at the same time. The blood samples were then collected 4 h later.

### 2.4. Biochemical Determination

Serum was obtained by cardiac puncture under anesthesia 1 h after drug treatment and was centrifuged (3000× *g*, 10 min, 4 °C). Urine supernatants were collected after centrifugation (3000× *g*, 10 min) for analysis. UA and creatinine (CR) levels were all measured using commercial kits (BioAssay Systems, Hayward, CA, USA).

### 2.5. Determination of MDA and GSH Concentrations and SOD Activity in Kidneys

The kidney tissues were homogenized, and the concentration levels of the proteins were determined by the BCA Kit (Tiangen Biotechnology; Beijing, China). The MDA and GSH contents and SOD activity were assessed using the MDA Assay Kit (Solarbio, Beijing, China), Cellular GSH Assay Kit, and the Total SOD Assay Kit (Beyotime Biotechnology, Shanghai, China).

### 2.6. Histopathological Analysis

The kidneys were decalcified in 4% paraformaldehyde and stained with hematoxylin-eosin (H&E) and Masson’s trichrome. The specimens were photographed using a light microscope using 200× magnification. The degree of renal tubular injury was assessed as reported in a previous study [36]. Briefly, a score of 0–4 was used to identify the tubular damage in the kidney (0: no damage, 1: <25% injury, 2: <50% injury, 3: <75% injury, and 4: >75% injury). The areas of collagen deposition were evaluated and graded by Image J software.

### 2.7. RT–qPCR

Total RNA from the renal cortex was extracted using the Total RNA Isolation Kit for Animal Tissue (Foregene, Chengdu, China). The quantity and quality of extracted RNAs within each group were detected via absorbance at 260 nm and 280 nm using a NanoDrop NC2000 spectrophotometer (Thermo Fisher Scientific, Waltham, MA, USA). Then, PrimeScript™ RT Master Mix (Takara, Japan) was applied to obtain the cDNA samples. The qPCR components contained 5 μL of Power SYBR Green Master Mix (Thermo Fisher Scientific, USA), 1 μL of cDNA (500 ng/μL), and 0.5 μL (10 μM) for both the forward and reverse primers and 3 μL of ddH_2_0. The qPCR experiment was then carried out with Roche LightCycler 480II (Roche, Basel, Switzerland). All the primer sequences used in the present study are listed in Table S3, and β-actin was chosen as the endogenous control. The relative expression levels of the targeted genes were then calculated by the 2^−ΔΔCt^ method.

### 2.8. Western Blot

Briefly, the tissue proteins were obtained and quantified after homogenizing in RIPA buffer containing protease and phosphatase inhibitor (NCM Biotech, Suzhou, China). An amount of 40 μg of protein lysate was resolved by SDS–PAGE gels, and the protein was then transferred onto PVDF membranes. The membranes were blocked in 5% skim milk, followed by incubation with the primary antibodies URAT1 (1:1000; Absin; Shanghai, China), GLUT9 (1:2000; LSBio, Seattle, WA, USA), or GAPDH (1:5000; Signalway Antibody, Greenbelt, MD, USA) overnight at 4 °C. Finally, the protein bands were visualized using a multifunctional imaging analysis system (FluorChem R ProteinSimple, Minneapolis, MN, USA) and quantified with intensities by ImageJ software.

### 2.9. Gut Microbiota Analysis

Fecal samples from the mice were freshly frozen in liquid nitrogen and then stored at −80 °C until analysis. Total genomic DNA was extracted using the E.Z.N.A.^®^ soil DNA Kit (Omega Bio-Tek, Norcross, GA, USA). The DNA concentration was detected by a NanoDrop NC2000 spectrophotometer and confirmed by agarose gel electrophoresis.

The bacterial 16S rRNA gene V3-V4 region was amplified with the primer pair 338F (5′-ACTCCTACGGGAGGCAGCAG-3′) and 806R (5′-GGACTACHVGGGTWTCTAAT-3′). PCR amplification was performed on an ABI GeneAmp^®^ 9700 PCR thermocycler (ABI, Los Angeles, CA, USA). The DNA samples were then sequenced by using the Illumina Miseq sequencer PE250 (Illumina, San Diego, CA, USA). 

The microbial compositions and abundance were visualized and analyzed using QIIME 2.0, as described in a previous study [37]. The Kruskal–Wallis H test was applied to detect species that exhibited differences in abundance of the microbial compositions in each group. The correlation of the gut microbiota composition with the HN phenotype and SCFAs concentration was analyzed by Spearman’s correlation analysis; the correlations were considered significant when *p* < 0.05.

### 2.10. GC–MS Analysis of the SCFAs Concentrations

SCFAs in fecal samples were examined by GC–MS, according to a previous report with a few modifications [38]. Briefly, 200 mg of fecal samples were mixed with 2 mL pure water and fully vortexed for 5 min before centrifugation (8000× *g*, 10 min). Afterward, 1.5 mL of supernatant was added with 150 μL of sulfuric acid (50%) and 2.5 mL of ether and centrifuged (8000× *g*, 10 min) again after extraction for 5 min. Then, 100 μL of internal standard (2-ethylbutyric acid, 500 μg/mL, Sigma–Aldrich) was added to 1 mL of the final supernatant in ether. Finally, the samples were loaded into GC–MS after filtering through 0.22 μm filters.

### 2.11. Cell Culture and Treatment

HEK-293T cells were cultured with DMEM containing 10% FBS (Gibco; Billings, MT, USA) in an incubator (37 °C with 5% CO_2_). The plasmids (URAT1 or GLUT9) were transfected into the HEK-293T cells using Lipofectamine 3000 (Invitrogen, Carlsbad, CA, USA) using 24-well culture plates, respectively. After 24 h, the cells were used for ^14^C-urate transport assays and electrophysiological recordings via the whole-cell patch clamp technique to explore the effects of SCFAs on the urate transport activity of URAT1 and GLUT9 were performed, as per the previous studies described by us [39,40].

### 2.12. Molecular Docking

The protein crystal structures of URAT1 and GLUT9 were built by the swiss-model (https://swissmodel.expasy.org/, accessed on 1 September 2021). The Protein Preparation Wizard module in Schrodinger Maestro was taken into consideration for the protein structure treatment, which included removing water and ions, protonation, adding missing atoms, completing missing groups, minimizing protein energy, and optimizing energy.

Molecular docking was accomplished using the Glide module. The receptor performed preprocessing, optimization, and minimization (constrained minimization using the OPLS3e force field). Acetic acid, propionic acid, and butyric acid were prepared according to the default settings of the Ligpre module. The prepared receptors were imported into the receptor grid generation when the screening in the Glide module was conducted. The key residues were then selected as the center of the box. Finally, the XP module with high precision was used for molecular docking to correlate pose and for better scores.

### 2.13. Statistical Analysis

Statistical analysis was performed with GraphPad Prism 8.0 Software (GraphPad Software, San Diego, CA, USA). All data are presented as the mean ± SD. One-way ANOVA with Dunnett’s multiple comparison test was used for statistical significance between groups except for the analysis of the microbiota data. Differences with *p* < 0.05 were considered statistically significant.

## 3. Results

### 3.1. Effect of BL on Physiological and Biochemical Indexes in Mice with HN

**Figure 1:**
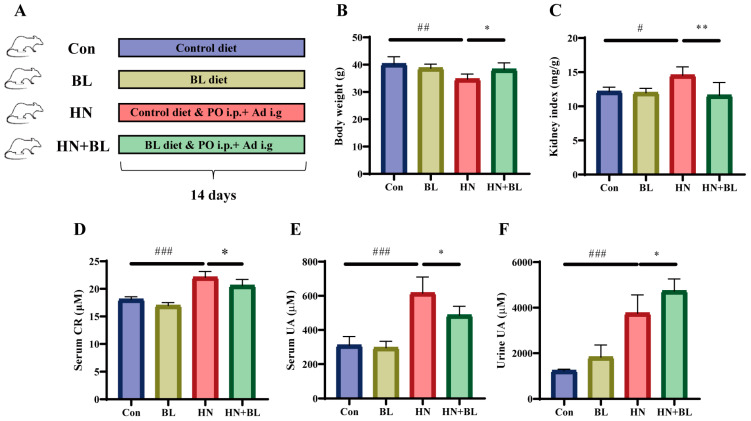
Effect of barley leaf (BL) on the physiological and biochemical indexes in mice with HN. (**A**) Experimental schematic of BL treatment. (**B**) Body weight. (**C**) Kidney index. (Kidney index = kidney weight (mg)/body weight (g)). (**D**) Serum CR. (**E**) Serum UA. (**F**) Urine UA level. Data represent mean ± SD (*n* = 6). # *p* < 0.05, ## *p* < 0.01, ### *p* < 0.001 vs. the Con group; * *p* < 0.05, ** *p* < 0.01 vs. the HN group.

A schematic diagram for the establishment of the HN mouse model and BL treatment is illustrated in Figure 1A. The food intake of the mice in each group showed no significant difference (Figure S1). After 14 days of PO and Ad intervention in the mice, the HN model group showed obvious body weight loss and kidney index gain (Figure 1B,C). The levels of serum CR (Figure 1D), serum UA (Figure 1E), and urine UA (Figure 1F) were all significantly elevated in those mice with HN. After the administration of BL, the body weight and the kidney index were similar to those of the normal mice, and the levels of serum CR and UA had markedly decreased. At the same time, UA excretion in the urine further increased. Meanwhile, BL had no significant effects on the control mice.

### 3.2. BL Ameliorated Kidney Damage in Mice with HN

**Figure 2:**
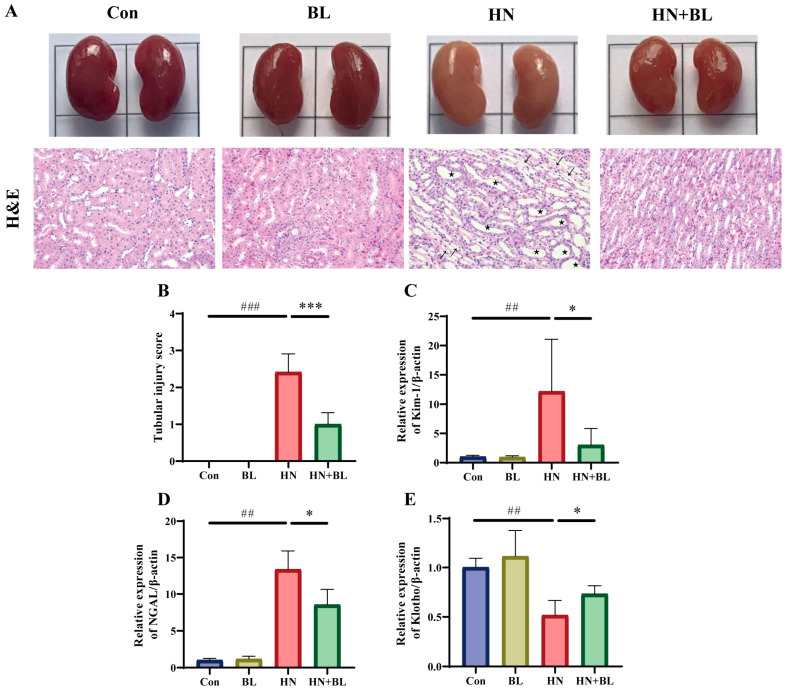
Effect of BL on renal histopathology in mice with HN. (**A**) The appearance and the H&E staining photomicrographs of the kidneys. (**B**) The semiquantitative scoring of the tubular injury in the H&E-stained kidney slides. (**C**–**E**) The relative mRNA expression levels of early renal injury biomarkers Kim-1, NGAL, and Klotho. Pentagon: renal tubule dilatation. Arrow: inflammatory cell infiltration. Data represent mean ± SD (*n* = 6). ## *p* < 0.01, ### *p* < 0.001 vs. the Con group; * *p* < 0.05, *** *p* < 0.001 vs. the HN group.

To demonstrate the protective effects of BL on the kidneys in the mice with HN, their kidney pathology and renal fibrosis were observed. As revealed in Figure 2A, the kidneys of the mice with HN were dramatically swollen in their macroscopic characteristics. H&E staining of the kidney also showed renal damage with obvious tubular epithelial cell edema, renal tubule dilatation, and inflammatory cell infiltration. In contrast, the BL treatment group not only recovered from the swelling but also ameliorated the renal pathomorphological damage. Moreover, the two kidney injury markers, namely kidney injury molecule-1 (Kim-1) and neutrophil gelatinase-associated lipocalin (NGAL) were distinctly greater in the mice with HN at the gene level, while these were reversed by the BL treatment (Figure 2C,D). Furthermore, BL administration markedly increased the expression of Klotho (Figure 2E), which was detected as being reduced when kidney damage occurred. The data suggested that BL could effectively ameliorate renal damage.

### 3.3. BL Alleviated Renal Fibrosis in Mice with HN

**Figure 3:**
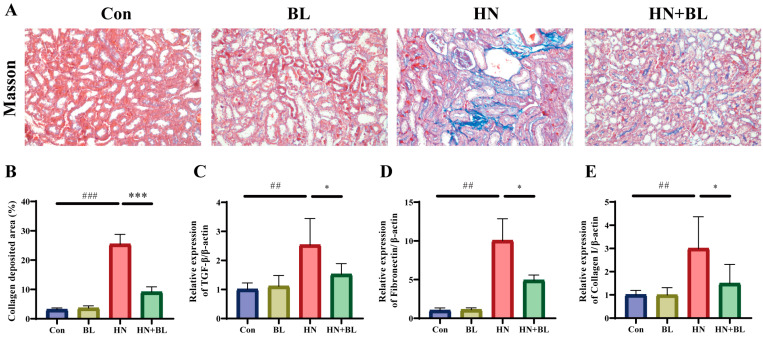
Effect of BL on the renal fibrosis in mice with HN. (**A**,B) The photomicrographs and analysis of the collagen deposition area of Masson’s trichrome staining. (**C**–**E**) The relative mRNA expression of renal TGF-β1, Fibronectin and Collagen I. Data represent mean ± SD (*n* = 6). ## *p* < 0.01, ### *p* < 0.001 vs. the Con group; * *p* < 0.05, *** *p* < 0.001 vs. the HN group.

As is shown by Masson staining (Figure 3A,B), the blue staining area in the renal tubulointerstitial indicated collagen accumulation, which was clearly observed in the HN group. However, BL could attenuate the renal collagen accumulation effectively. Transforming growth factor-β1 (TGF-β1), which can be produced by macrophages, is regarded to be a critical marker in the generation and progression of renal fibrosis, and Fibronectin and Collagen I are also important biomarkers of fibrosis [41]. The relative mRNA expression levels of TGF-β1, Fibronectin, and Collagen I in mice from the HN group were significantly enhanced compared to those in the Con group, and the BL diet notably suppressed the upregulation (Figure 3C–E).

### 3.4. BL Reduced Renal Oxidative Stress in Mice with HN

**Figure 4:**
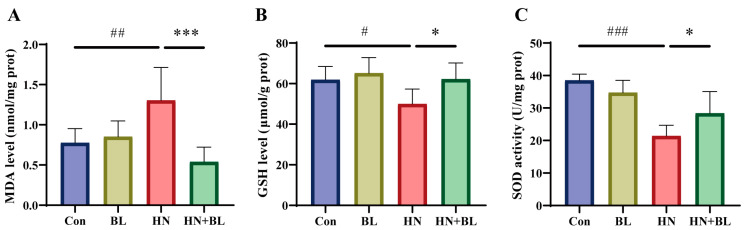
Effects of BL on oxidative stress in the kidneys of mice with HN. The levels of MDA (**A**), GSH (**B**), and SOD activity (**C**) in the kidneys. Data represent mean ± SD (*n* = 6). # *p* < 0.05, ## *p* < 0.01, ### *p* < 0.001 vs. the Con group; * *p* < 0.05, *** *p* < 0.001 vs. the HN group.

MDA is an end product of lipid peroxidation, and excessive lipid peroxidation causes the depletion of the antioxidant glutathione (GSH). SOD is one of the antioxidant defense systems for eliminating ROS. As displayed in Figure 4, the MDA content had markedly increased, whereas both the GSH content and the SOD activity decreased in the HN group. BL intervention markedly reduced the level of MDA and increased the level of GSH and the activity of SOD when compared to the HN group. The results demonstrated that the administration of BL could alleviate renal oxidative stress in mice with HN.

### 3.5. BL Suppressed the Protein Expression Levels of Renal URAT1 and GLUT9 in Mice with HN

**Figure 5:**
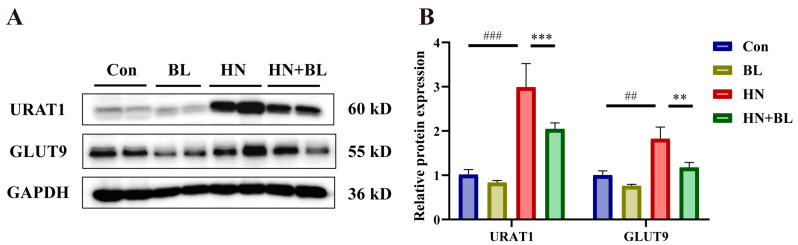
Effects of BL on the relative protein expression levels of renal URAT1 and GLUT9 in mice with HN. (**A**) The protein bands. (**B**) Renal URAT1 and GLUT9 protein levels were normalized to GAPDH. Data represent mean ± SD (*n* = 4). ## *p* < 0.01, ### *p* < 0.001 vs. the Con group; ** *p* < 0.01, *** *p* < 0.001, vs. the HN group.

As is revealed in Figure 5, PO and Ad intervention markedly raised the protein expression levels of renal URAT1 and GLUT9. However, BL dramatically downregulated their expression in the mice with HN, which is also evident by the increase in urine UA levels in Figure 1F.

### 3.6. BL Increased the Contents of Fecal SCFAs in Mice with HN

**Figure 6:**
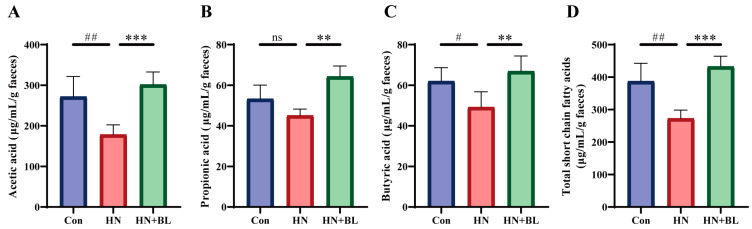
Effect of BL on the fecal SCFAs content in mice with HN. (**A**) Acetic acid. (**B**) Propionic acid. (**C**) Butyric acid. (**D**) Total SCFAs. Data represent mean ± SD (*n* = 6). # *p* < 0.05, ## *p* < 0.01, vs. the Con group; ** *p* < 0.01, *** *p* < 0.001 vs. the HN group; ns means no significance.

In this study, the concentrations of acetic acid, propionic acid, and butyric acid were evaluated by GC–MS. As is shown in Figure 6, PO and Ad administration for two weeks led to significant decreases in acetic acid, propionic acid, butyric acid, and total SCFAs levels. In contrast to the HN group, BL treatment remarkably increased the levels of acetic acid, propionic acid, butyric acid, and total SCFAs.

### 3.7. BL Altered the Structure of the Intestinal Flora in Mice with HN

**Figure 7:**
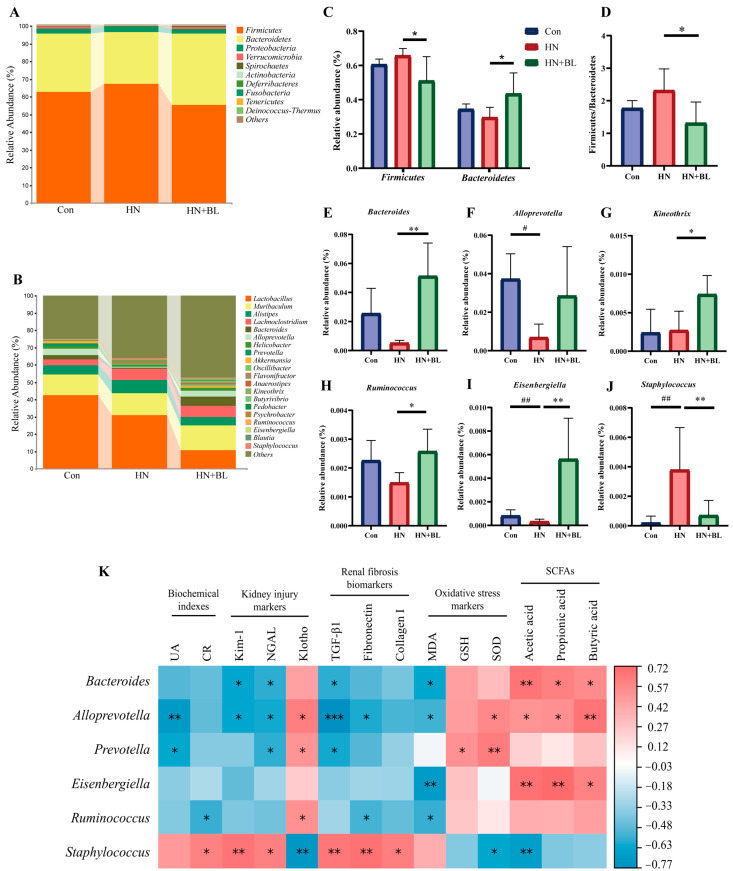
Effect of BL on the gut microbiota composition in the mice with HN. (**A**,**B**) Regulated microbial compositions at the phylum level and the genus level. (**C**) The relative abundance of Firmicutes and Bacteroidetes at the phylum level. (**D**) *Firmicutes/Bacteroidetes* (F/B) ratio. (**E**–**J**) The relative abundance of *Bacteroides*, *Alloprevotella*, *Kineothrix*, *Ruminococcus*, *Eisenbergiella*, and *Staphylococcus*. Data represent mean ± SD (*n* = 5). # *p* < 0.05, ## *p* < 0.01 vs. the Con group; * *p* < 0.05, ** *p* < 0.01, vs. the HN group. (**K**) Spearman’s analysis of bacterial abundance with the determined measurements, including serum UA and CR, the renal mRNA expression of Kim-1, NGAL, Klotho, TGF-β, Fibronectin and Collagen I, MDA and GSH content, and SOD activity in the kidneys, as well as the SCFAs concentrations. (* *p* < 0.05, ** *p* < 0.01, *** *p* < 0.001 with r > 0.5 or r < −0.5.).

16S rRNA gene sequencing of the gut microbiota from the fecal samples was performed to analyze the composition of the gut microbial communities. Specific differences in gut microbiota abundance were observed. The results showed that the predominant bacterial species at the phylum level were mainly *Firmicutes* and *Bacteroidetes*, but the proportions changed throughout all groups (Figure 7A). In the HN group, the relative abundance of *Firmicutes* and *Bacteroidetes* and the ratio of *Firmicutes/Bacteroidetes* (F/B) slightly shifted. Compared with the HN group, the relative abundance of *Firmicutes* was lower (Figure 7C) in the HN + BL group, whereas the relative abundance of *Bacteroidetes* and the ratio of F/B was significantly higher (Figure 7D). At the genus level (Figure 7B), the structure of the intestinal flora also shifted in the HN group compared with the Con group, as evidenced by the reduction in the abundance of *Bacteroides*, *Alloprevotella*, *Kneothrix*, *Ruminococcus*, and *Eisenbergiella* and enhanced *Staphylococcus*. Inversely, BL significantly elevated the abundance of *Bacteroides*, *Alloprevotella*, *Kineothrix*, *Ruminococcus*, and *Eisenbergiella*, while reducing the abundance of *Staphylococcus* in the mice with HN (Figure 7E–J).

Furthermore, Spearman’s correlation analysis was performed to correlate the composition of the gut microbial communities with biochemical indexes (UA and CR), kidney injury markers (Kim-1, NGAL, and klotho), renal fibrosis biomarkers (TGF-β1, Fibronectin and Collagen I), oxidative stress markers (MDA, GSH, and SOD), and fecal SCFAs concentration. Figure 7K showed that *Bacteroides, Alloprevotella*, *Prevotella*, *Eisenbergiella*, and *Ruminococcus* were generally negatively correlated with the HN phenotype, while *Staphylococcus* was positively correlated with these markers. Besides, *Bacteroidetes*, *Alloprevotella*, and *Eisenbergiella* were found to be significantly positively associated with the fecal levels of acetic acid, propiontic acid, and butyric acid.

### 3.8. SCFAs Reduced Serum UA Levels In Vivo and Inhibited the Urate Transport Activities of URAT1 and GLUT9

**Figure 8:**
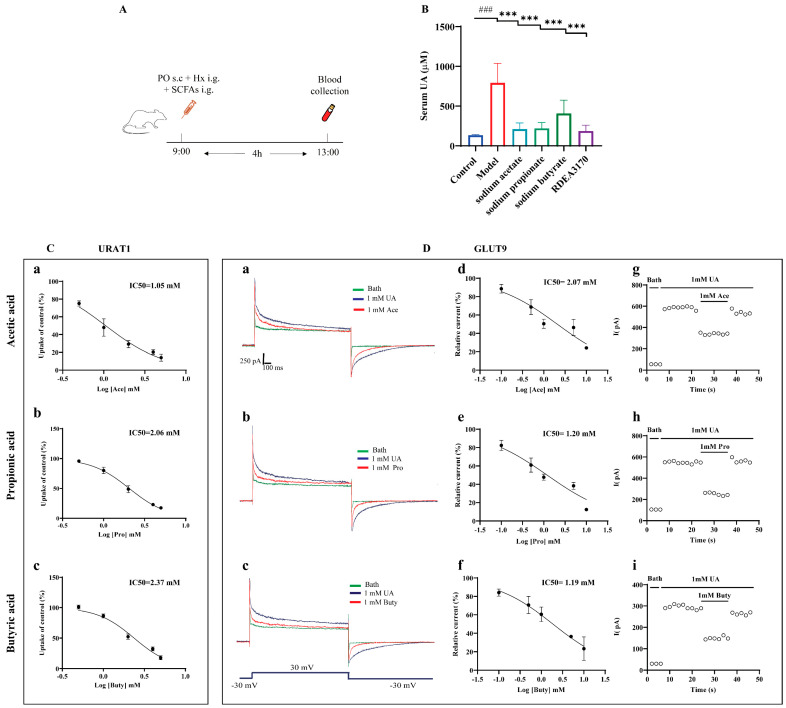
Effect of SCFAs on serum UA levels in PO + Hx-induced hyperuricemic mice and URAT1- and GLUT9-mediated urate transport in over-expressing HEK293T cells. (**A**) Experimental schematic of SCFAs treatment. (**B**) Serum UA (*n* = 6). (**C**) Effects of acetic acid (**a**), propionic acid (**b**), and butyric acid (**c**) on the transport activity of URAT1 (*n* = 6). (**D**) Effects of acetic acid, propionic acid, and butyric acid on GLUT9-mediated urate transport: (a–c) original current traces of GLUT9-expressing HEK-293T cells induced by 1 mM UA in the presence (red) or absence (blue) of 1 mM acetic acid, propionic acid, and butyric acid; (d–f) dose-dependent inhibition of acetic acid, propionic acid, and butyric acid on GLUT9; (g–i) time-course of current with the perfusion of 1 mM acetic acid, propionic acid, and butyric acid after stimulation with 1 mM UA (*n* = 6). Data are indicated as mean ± SD. ### *p* < 0.001, vs. the Control group; *** *p* < 0.001 vs. the Model group.

Concerning the potential relationship between SCFAs and HUA, we further evaluated whether the administration of SCFAs prevented abnormal UA metabolism in mice with HUA, according to the protocol indicated in Figure 8A. The results demonstrated that an increased serum UA level was greatly reversed by SCFAs treatment in the PO + Hx-induced mice with HUA (Figure 8B). 

Next, the effects of the SCFAs on the urate transport activities of URAT1 and GLUT9 in vitro was investigated. Both URAT1 and GLUT9 mainly mediate urate reabsorption in the kidneys. URAT1 completes the reabsorption of urate through the exchange of various organic anions, while GLUT9 is an electrogenic and voltage-dependent transporter. Therefore, the urate transport activities of URAT1 and GLUT9 were detected by ^14^C-urate transport assays and electrophysiological recordings, respectively. The results showed that acetic acid, propionic acid, and butyric acid dose-dependently inhibited URAT1 (Figure 8C) and GLUT9 (Figure 8D(d–f)), with IC50 values of 1.05 mM, 2.05 mM, and 2.37 mM for URAT1 and 2.07 mM, 1.20 mM, and 1.19 mM for GLUT9, respectively. Further experiments found that the inhibitions of GLUT9 by the SCFAs can be eluted (Figure 8D(g–i)), implying that the inhibitions of GLUT9 by SCFAs are reversible, similar to probenecid, as we previously reported [38].

### 3.9. Molecular Docking Analysis

**Figure 9:**
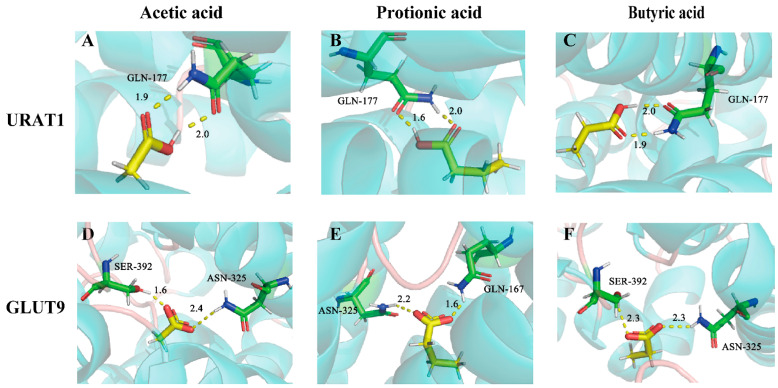
The binding modes of acetic acid, propionic acid, and butyric acid with URAT1 (**A**–**C**) and GLUT9 (**D**–**F**).

Molecular docking was also applied to explore the potential binding modes of SCFAs with URAT1 and GLUT9. As is displayed in Table S4, these compounds all exhibited intense binding energies with URAT1 and GLUT9, which were –4.61 kcal/mol, −6.13 kcal/mol, and −6.25 kcal/mol for URAT1 and −3.78 kcal/mol, −5.00 kcal/mol, and −4.98 kcal/mol for GLUT9, respectively. According to the binding mode, the compound and the amino acid residues (GLN-177) (Figure 9A–C) of the protein pocket of URAT1 or the amino acid residues (SER-392, ASN-325, GLN-167) of the protein pocket of GLUT9 could be seen clearly (Figure 9D–F). The carboxyl group of each compound can form multiple hydrogen bonds with the amino acids of URAT1 or GLUT9. For example, the carboxyl group of acetic acids and the active group of amino acids form two strong hydrogen bonds, with distances of 1.9 Å and 2.0 Å for URAT1 and 1.6 Å and 2.4 Å for GLUT9.

## 4. Discussion

UA is the end product of purines, and the excessive intake of diets that are rich in purines might contribute to increased serum UA [5]. Chronically elevated UA levels often cause kidney injuries, including renal inflammation and fibrosis, and may further develop into HN. Current therapy for HUA mainly depends on the application of XO inhibitors and uricosuric agents, which might cause various side effects, such as kidney damage or fulminant hepatitis, when under long-term use [14]. Consequently, more novel and effective treatments for HN need to be investigated.

BL has been a popular functional food due to its powerful and excellent health advantages on gut functioning [42]. However, the preventative role of BL on HN has not been explored. In the present study, we found the critical role that BL plays in a mouse model with HN induced by PO and Ad. Our data demonstrate that a dietary supplement of BL can remarkably decrease serum UA levels and also ameliorate renal damage and renal fibrosis, which was possibly related to the modulation of the intestinal flora structure and the metabolite SCFAs.

Excessive UA can lead to the oxidation of DNA, proteins, lipids, cell apoptosis, and organ dysfunction, all of which will activate the immunological inflammatory system and cause oxidative stress, eventually leading to renal damage, including nephritis and fibrosis [43]. As was shown in the kidneys of the mice with HN, severe renal tubule dilatation and collagen deposition were detected, which were all ameliorated after BL treatment. Many researchers [44,45,46,47] have found that many dietary supplements exert renal protective effects and UA-lowering properties due to their antioxidant and anti-inflammatory effects. Based on our data, BL also played a role in modulating the oxidative stress indexes in the kidneys, thus improving renal dysfunction.

Urate transporters are key targets in regulating UA homeostasis. URAT1 and GLUT9, which mediate the exchange of intraluminal UA with inorganic anions (Cl-) and organic anions (lactate and hydrochloride) in proximal tubule epithelial cells, are critical transporters for UA reabsorption from the kidney lumen to the blood [10]. URAT1 and GLUT9 reabsorb almost 90% of the excreted UA [6]. Thus, many novel uricosuric drugs and candidates, such as lesinurad, RDEA3170, and CDER167 are designed to specifically target URAT1 and GLUT9 by inhibiting their functions [12]. Many previous studies have reported that dietary supplements play a role in reducing UA by inhibiting the expression of URAT1 or GLUT9 [44,48,49]. However, they rarely studied whether the UA-lowering activities are related to the changes in the urate-transporting activities of URAT1 and GLUT9. In this study, we also found that BL notably reversed the increases in renal URAT1 and GLUT9 expression levels in mice with HN. More interestingly, we also explored the effects of BL on the urate-transporting activities of URAT1 and GLUT9 in vitro. The results indicated that BL did not affect URAT1 and GLUT9, even at 1 mg/mL (shown in Figure S2), which motivated us to believe that the anti-hyperuricemic effect of BL may be due to gut microbiota regulation or its metabolites.

Growing evidence suggests that the gut microbiota is closely connected to various diseases, including colitis, diabetes, HUA, gout, and so on [50]. Dietary fiber is recognized to play a critical role in preventing chronic diseases by modulating the composition of intestinal microbiota and promoting the formation of various endogenous metabolites, such as SCFAs [51,52,53]. A recent study reported that BL could attenuate colitis and colorectal cancer by regulating intestinal flora [29,30,31]. On the one hand, we found that a supplement of BL significantly regulated the SCFAs-producing bacteria and the fecal SCFAs contents in HN mice. On the other hand, BL significantly altered the gut bacterial compositions at both the phylum and the genus levels. BL reduced the abundance of *Staphylococcus* and enhanced the richness of *Bacteroides*, *Alloprevotella*, *Kneothrix*, *Ruminococcus*, and *Eisenbergiella*. *Bacteroides* [52,54], *Alloprevotella* [55], and *Eisenbergiella* [56], the well-known SCFAs-producing bacteria, showed a significant positive correlation with SCFAs in this study. Additionally, *Kineothrix* is a butyrate-producing bacterium belonging to the *Lachnospiraceae* family [57]. *Ruminococcus*, the abundance of which decreased in *Uox*-knockout mice, is also known as a SCFAs-producer [22]. *Staphylococcus* is known as a pathogenic microorganism that increases the risk of pathogenic invasion and intestinal inflammation [58]. In the current study, *Staphylococcus* showed a significant positive correlation with the kidney injury markers and renal fibrosis biomarkers, indicating that it might play a role in HN progression. These changes in the compositions might help explain the beneficial effects of BL on HN-related kidney injury. On the other hand, fecal SCFAs levels were markedly enhanced after BL treatment.

SCFAs are considered the key metabolites of gut microbes that affect host health [59]. They can pass through the “gut-renal axis” to reach the body’s kidneys and play a role in kidney protection [60]. In recent years, studies have widely implicated that the abundance of SCFAs-producing bacteria in HUA mice has been reduced [22,61] and that butyrate synthesis in the feces of gout patients was also significantly reduced [19], suggesting that there may be a certain relationship between SCFAs and the level of UA in the body. Moreover, many previous studies have shown that probiotics (*Lactobacilli* and *Bifidobacteria*) and prebiotics (phytochemicals, polyphenols, and peptides) exert UA-lowering effects by altering the intestinal environment and raising the abundance of SCFAs-producing bacteria [23]. However, these studies did not propose whether the above effects are directly related to SCFAs. Hence, we further explored the UA-lowering effects of SCFAs in hyperuricemic mice. The results showed that the administration of sodium acetate, sodium propionate, and sodium butyrate could significantly reduce serum UA levels. SCFAs enter the cells primarily through transporters (SMCT1/2 and MCT1/4) in colonic epithelial cells [62]. SMCT1 is expressed in not only the intestinal tract but also the kidneys. It can transport lactic acid and promote the exchange of lactate and urate [63]. Therefore, we wondered whether SCFAs could also affect the urate transport process of URAT1 and GLUT9. Of interest, the data in Figure 8 revealed that SCFAs potently dose-dependently inhibit URAT1 and GLUT9 in vitro. Based on the electrophysiological experiments, the inhibitory activity of SCFAs on GLUT9 was reversible. Furthermore, the molecular docking results also showed that acetic acid, propionic acid, and butyric acid could bind with URAT1 and GLUT9 (Figure 9).

Our present study successfully demonstrated the direct effects of SCFAs on the functions of URAT1 and GLUT9 in vitro. However, SCFAs are considered to function by activating G-protein membrane receptors, like GPR41 and GPR43, or inhibiting histone deacetylases (HDACs) [64]. HDAC inhibition has a wide array of downstream effects, including regulating gene expression [65]. Moreover, GPR41 and GPR43 have been proven to play significant roles in regulating metabolism, inflammation, and other diseases. Thus, further investigation is needed to explore whether the beneficial effects of SCFAs on HUA involve HDAC inhibition or GPCR activation.

## 5. Conclusions

When taken together, the results of our study reveal that dietary BL supplementation can protect kidneys from HUA-associated damage, which may be attributed to its antioxidant and antifibrotic effects and its regulation of gut microbiota composition, as well as the enhancement of fecal SCFAs production. More importantly, SCFAs were first demonstrated to exhibit potential antihyperuricemic effects by inhibiting URAT1 and GLUT9 in vitro. In total, these findings suggest that BL might be a potential dietary supplement for the prevention of HUA or HN.

## Data Availability

The data presented in this study are available on request from the corresponding author.

## Supplementary Materials

The following supporting information can be downloaded at: https://www.mdpi.com/article/10.3390/foods11213482/s1, Table S1: The macronutrient composition of the BL powder in the present study; Table S2: The composition of the CD and BL diets; Table S3: Primers for RT-qPCR analysis; Table S4: The binding energies of SCFAs with URAT1 and GLUT9. Figure S1: The food intake of mice. Figure S2: Effect of BL on URAT1 and GLUT9-mediated urate transport in vitro.

## Abbreviations

HUA: hyperuricemia; UA, uric acid; hyperuricemic nephrology, HN; barley leaf, BL; SCFAs, short-chain fatty acids; URAT1, urate transporter 1; GLUT9, glucose transporter 9; KIM-1, kidney injury molecule-1; NGAL, neutrophil gelatinase-associated lipocalin; TGF-β1, transforming growth factor-β1; MDA, malondialdehyde; GSH, glutathione; SOD, superoxide dismutase.
