# Supplementary material for: Insoluble Fiber in Barley Leaf Attenuates Hyperuricemic Nephropathy by Modulating Gut Microbiota and Short-Chain Fatty Acids
Source: Foods. 2022 Nov 2;11(21):3482. doi: 10.3390/foods11213482 (PMC9656210; doi:10.3390/foods11213482)
Supplement: Supplementary file 1 [file foods-11-03482-s001.zip › foods-1912287-supplementary.pdf]

Table S1. The macronutrient composition of the BL powder in the present study.

Item	Unit (g/100g)
Protein	23.9
Fat	2.98
Carbohydrate	62.3
Soluble dietary fiber	< 0.01
Insoluble dietary fiber	53.6
Moisture	3.99
Ash	6.8

Table S2. The composition of CD and BL diet.

Ingredients (g/kg of diet)	Diets	
	CD	BL
Barley leaf powder	0	25
Casein	189.58	178.58
L-Cysteine	2.84	2.84
Corn Starch	298.59	280.59
Maltodextrin	33.18	33.18
Sucrose	331.77	327.77
Cellulose	47.40	47.40
Soybean oil	23.70	23.70
Lard	18.96	18.96
Mineral Mix M1002	9.48	9.48
DiCalcium Phosphate	12.32	12.32
Calcium Carbonate	5.21	5.21
Potassium Citrate	15.64	15.64
Vitamin mix V10001	9.48	9.48
Choline Bitartrate	1.90	1.90
Total	1000	1000

All diets are isocaloric and contained 19.2% protein, 4.3% fat and 67.3% carbohydrate. And the BL diets contained 1.34% insoluble dietary fiber.

Table S3. Primers for RT-qPCR analysis.

Gene	Forward Primer Sequence (5′→3′)	Reverse Primer Sequence (5′→3′)
Kim-1	ACATATCGTGGAATCACAACGAC	ACTGCTCTTCTGATAGGTGACA
NGAL	AATGTCACCTCCATCCTGGT	ATTTCCCAGAGTGAACTGGC
Klotho	TCTCAAGAAGTTCATAATGGAAACC	CAGAAAGTCAACGTAGAAGAGTCCT
TGF-β1	CCACCTGCAAGACCATCGAC	CTGGCGAGCCTTAGTTTGGAC
Fibronectin	CCCTATCTCTGATACCGTTGTCC	TGCCGCAACTACTGTGATTCGG
Collagen I	CCTCAGGGTATTGCTGGACAAC	CAGAAGGACCTTGTTTGCCAGG
β-actin	AAGTCCCTCACCCTCCCAAAAG	AAGCAATGCTGTCACCTTCCC

Table S4. The binding energies of SCFAs with URAT1 and GLUT9.

Name	Compound structure	Binding energy with URAT1(kcal/mol)	Binding energy with GLUT9(kcal/mol)
Acetic acid	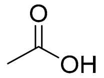	-4.61	-3.78
Propionic acid	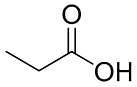	-6.13	-5.00
Butyric acid	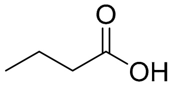	-6.25	-4.98

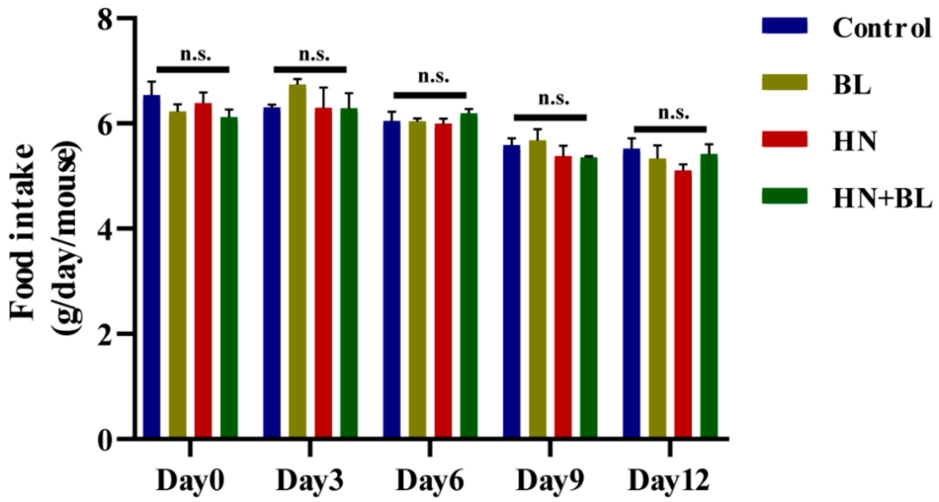

Figure S1. The food intake of the mice in each group on Day 3/6/9/12. Data are presented as the mean ± SD. n.s. means no significant.

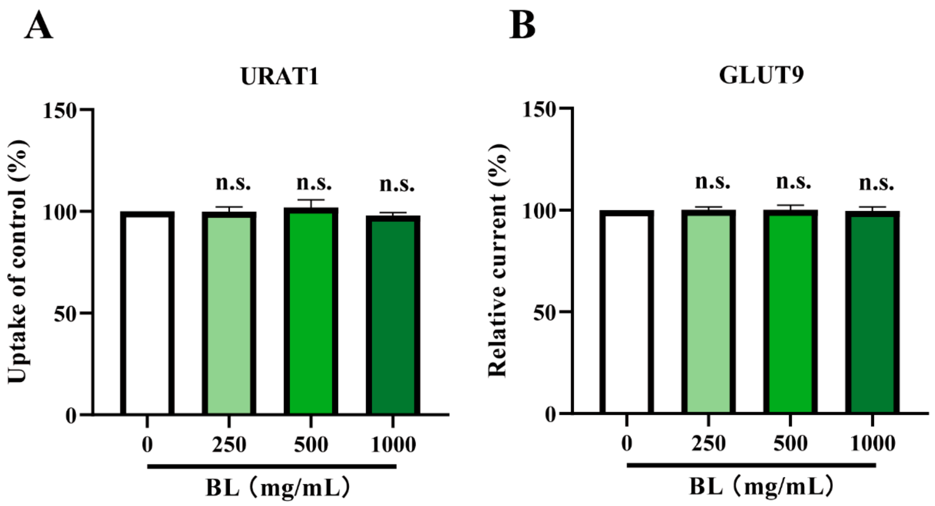

Figure S2. Effect of BL on URAT1 and GLUT9-mediated urate transport in vitro. (A) The relative uptake of ^{14}C-uric acid with perfusion of BL (0, 250, 500, 1000 mg/mL) on URAT1-mediated urate transport (n = 6). (B) The relative current with perfusion of BL (0, 250, 500, 1000 mg/mL) after stimulation with 1 mM UA on GLUT9-mediated urate transport (n = 6). Data are presented as the mean ± SD. n.s. means no significant.
